# Technical efficiency and its determinants in regulating adolescents’ coronavirus infection across Asian countries

**DOI:** 10.1038/s41598-023-45442-3

**Published:** 2023-11-01

**Authors:** Shrabanti Maity, Anup Sinha

**Affiliations:** 1 Department of Economics, Vidyasagar University, Midnapore, West Bengal India; 2 Department of Commerce, Karimganj College, Karimganj, Assam India

**Keywords:** Diseases, Health care

## Abstract

The coronavirus pandemic, besides generating health distress, influences the socio-economic conditions of humankind. Every adolescent's lifestyle is affected by the virus. Healthy adolescents are not only key contributors to the forthcoming workforce but also a source of a country’s human capital. The purpose of the article is to examine the efficacy of various Asian countries in regulating the spread of the coronavirus among adolescents. In addition to that, the paper also attempts to pinpoint the prime causes of the inefficiency of a country in regulating the same. The paper also examines the behavioural changes among adolescents across Asian countries in pre-and-post pandemic times. In this context, the study identifies the impact of adolescents’ tobacco consumption, female political leadership, and accreditation on a country’s efficacy to regulate adolescents’ coronavirus infection. The study’s empirical analysis covers twenty-one Asian countries. By using the Panel Stochastic Production Frontier, the study concludes that Kazakhstan is the most efficient country and Afghanistan is the least efficient country on the list. The inefficiency effects estimates conclude that adolescents’ tobacco consumption decreases and good governance practices increase the efficiency of a country in regulating the spread of adolescent coronavirus infection. Additionally, the paper finds no significant behavioural changes among adolescents in pre-and-post pandemic times across Asian countries. The paper concludes with appropriate policy recommendations supported by empirical evidence. The paper also identifies its shortcomings and suggests potential future lines of inquiry.

## Introduction

After the identification of the first case of a novel coronavirus in Wuhan, China, it soon spread to practically every country on the globe. Consequently, it becomes a global disaster and affects every nation, counting Asian countries^1^. Among the Asian countries, India, Iran, South Korea, Turkey, and Vietnam are the worst-affected nations. Unlike any other virus that has emerged in the past, coronaviruses affect humankind in an enormous and drastic manner. It is remarked as the most severe global disaster experienced by people in the global context since World War II^2^. The virus, on the one hand, has severe health apprehensions and, on the other hand, brings enormous socio-economic challenges and casualties for the people. In fact, according to recent studies, the recession that emerged from the Corona pandemic was considered the worst of the century because it exacerbated the negative consequences of the 2009 recession^3,4^.

### Existing literature

The virus poses a dissimilar risk level for different age groups^5^. In reality, a person's age has a substantial role in Corona contamination. Older adults who were 60 years of age or older with pre-existing health abnormalities experienced greater risk than other groups. Contrarily, the risk of pandemic appeared to be minimal among children and adolescents. According to the WHO report^6^, only 6.3% of older children and adolescents were infected by the virus on a global scale, while only 0.1% died due to disease. These figures are significantly lower than those of other older age groups. Studies have identified numerous factors that have been linked to early adolescents' decreased incidence of Coronavirus. One of the key factors in young people and adolescents having lower virus risks than older people may be the absence of numerous health difficulties, including cancer, renal failure, obesity, and many other disorders^7^. Additionally, unlike older adults, these groups have a stronger and more adaptive immune system, which may reduce the potential risk of SARS-CoV-2^8^. Besides these factors, studies also confirm that children and adolescents have more sophisticated lymphocytes and natural killer cells to fight against the virus than older adults^8,9^. This may be another reason for the lower severity among this group.

Even though the severity of the coronavirus is less likely among children and adolescents (particularly in the absence of underlying illnesses), it still affects them in many different ways than other groups^10^. Firstly, the outbreak of coronavirus raises health emergencies and risks among this group (although of lesser magnitude)^11^. Secondly, lockdown and school closures associated with the pandemic not only restrict physical activities but also their normal lifestyle^12,13^. Lastly and most importantly, health emergencies and social distancing bring unprecedented change, which ultimately increases mental health problems^14,15^. Further, studies claim that the level of anxiety, depression, and substance misuse among adolescents has increased considerably at the time of the Corona pandemic around the globe^16,17^. According to these emerging studies, the fear of the virus, social isolation, the demands of the new method of schooling, etc., are among the significant causes of such a difficult state^18^.

In addition, the WHO report^6^, asserts that even if there is a reduction in the number of instances of viral contamination among adolescents, this result may still be the result of inadequate testing and a lack of general symptoms to detect infection cases in the aforementioned category. According to some claims, it is still feasible for them to get the virus even if their symptoms may not resemble those of other older age groups and instead appear to be a common cold or flu^19,20^. Understanding the virus's detrimental effects on health, an untreated single incident may raise the likelihood of chronic health problems. Additionally, they might also increase the risk of spreading the virus among people^10^. It should be noted that the spread of this lethal virus poses a long-term health hazard^21–24^. As a result, persons who contracted corona should continue taking their medications, and adolescents are no exception^25^. Although their death rate was minimal due to their high immunity level, adolescents were also the group that was most negatively impacted by the corona^26^. Adolescents' mental health and academic progress have been jeopardised because of school closures and family economic turmoil caused by the COVID-19 lockdown^24,27^. Due to their higher likelihood of being asymptomatic positive, adolescents' corona infection severity was understated^28^. However, adolescents needed a longer hospital stay to recuperate^28^. The majority of South Asian countries routinely reported coronavirus case counts, but they did not disclose the virus's effects, such as unstable mental health disorders. Therefore, the true effect of mental health issues linked to the coronavirus is yet unknown or may even be underreported^24^.

Comprehensive research has been conducted to comprehend the various difficulties relating to the Coronavirus and adolescents. Nevertheless, the efficacy of a nation in regulating coronavirus infection among adolescents is not appropriately addressed. Consequently, there exists a research gap that must be filled in order to develop effective health policies.

### Objectives, motivation and novelty of the study

Accordingly, the present study includes *three objectives* to explore. Initially, the behavioural changes among adolescents in selected Asian countries are explored before, during, and after the pandemic. Then the efficacy of selected Asian countries in regulating the spread of coronavirus among adolescents is examined. Finally, the study involves itself in unravelling the factors responsible for inefficiency in regulating the same. In this regard, the study explores the consequences of Female Political Participation, Good governance practices, and tobacco use among adolescents on a nation’s efficiency in regulating the spread of Coronavirus among adolescents. Notably, the behavioural transmission among the adolescents in selected Asian countries is explored based on two variables, viz., "adolescent suicidal mortality rate (per 100,000)" and "tobacco use in adolescents (%)". The choice of the variables to analyse behavioural changes among adolescents in selected Asian countries is strictly dictated by the availability of the data. The motivation for this investigation is twofold: firstly, adolescents are the future human capital of a country, and health and education are crucial components of that capital. The stage of adolescence is a special phase of human life for building a concrete foundation for excellent health. The extent to which a nation is effective in creating a good health foundation for its demographic dividend must thus be investigated. The present study is exactly doing the same by inquiring about the efficacy of a country in regulating coronavirus infection among adolescents. Secondly, although the coronavirus is a global catastrophe, the present inquiry considered only Asian countries. This is because after the identification of the virus in China, Asian countries, including India, Japan, Taiwan, Thailand, and Vietnam, were the epicenter of the first wave of the virus. Additionally, since most Asian countries followed similar socio-economic and political structures, it is intellectually justifiable to focus on the Asian continent. Furthermore, any empirical research on a pandemic may only be done once the data is available, or by the end of the pandemic. Such empirical analysis helps in understanding the aftermath of the pandemic and aids in the formulation of policies to handle any adverse circumstances and recover from the aftershock. Moreover, the identification of the determinants of inefficiency in regulating adolescent corona infection allows the concerned government to pinpoint the areas that require their attention in order to create a society that is fully developed. Adolescents are the foundation of the economy of the future and the main source of human capital, so every nation's policy must be adequate for their growth and welfare. This justifies conducting such an empirical investigation even after the pandemic has ended. Again, existing literature evidences that corona infestation results in long-term health hazard^21–24^. Therefore, even after recovery, it is crucial that the infected person be treated with care. Corona-affected individuals must also get routine health examinations, and adolescents are not an exception in this regard. Adolescents are considered to be the future human capital of any nation; therefore, maintaining good health is a prerequisite for their transformation from humans to human capital. Due to this problem, the current study is still absolutely pertinent even after the epidemic has ended.

The study is novel in various aspects. It is perhaps the first study to involve itself in exploring the relative efficacy of different Asian countries in regulating the spread of Coronavirus among adolescents. Moreover, the identification of the factors responsible for inefficiency in regulating the adolescent coronavirus also makes the study unique. Furthermore, the choice of variables for identifying the factors responsible for the inefficiency of a nation in regulating adolescent coronavirus spread also makes the study novel.

The study is structured as described:

Section "Materials and methodology" presents the data sources, variables, and econometric model to examine the objectives following a brief introduction and specification of the objectives. The empirical results are presented in section "Results". Section "Discussion" presents the social-demographic and economic ramifications of the research findings. Finally, in section "Conclusion, limitation and future study direction", the study comes to a conclusion, offers policy recommendations, identifies its limitations, and specifies its course of further research.

## Materials and methodology

The theoretical underpinnings of the stochastic frontier model are discussed in this section. Additionally, this section includes the data sources, variables, and econometric model needed to experimentally investigate the aforementioned aims.

### Theoretical foundation

The study aims to scrutinise the efficiency of selected Asian countries in controlling the spread of Corona among the adolescent population. The empirical analysis will be facilitated by Stochastic Frontier Analysis (SFA). Notably, there are well-accepted approaches concerning efficiency measurement, viz., parametric and non-parametric. The SFA is supposed to be a highly acclaimed parametric technique. Contrarily, Data Envelopment Analysis (DEA) is considered the best non-parametric method to measure efficiency. Both approaches have their strengths and weaknesses. DEA is preferred over AFA when price data is not well defined or a concrete output-input orientation cannot be established^29,30^. Accordingly, the DEA has wide applications in health economics, crime efficiency, etc^29,30^. However, if the output-input relation is well-defined, then SFA is preferred over DEA for several reasons, including the non-inclusion of "*statistical noise*", non-utilisation of all information, and the improper identification of the decision-making unit’s efficiency.

**Figure 1:**
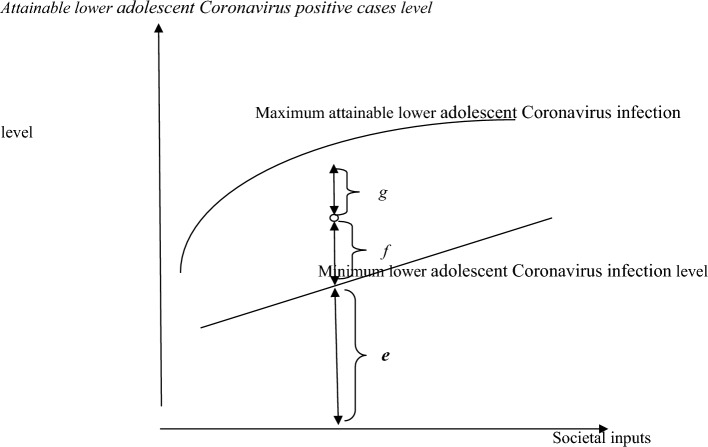
Performances in regulating adolescent Coronavirus infection. Source: Adapted from Murray and Frenk, (1999) and Evans, et al., (2000).

Initially, Evans et al.^31^ and Murray, and Frenk^32^ and afterward Sankar, and Kathuria^33^ and Kathuria and Sankar ^34^, successfully conceptualised the SFA approach to measure health system efficiency. The current paper follows Evans et al.^31^, Murray, and Frenk^32^, Sankar, and Kathuria^33^ and Kathuria and Sankar^34^, health efficiency concepts. Thus, the Stochastic Production Frontier (SPF) is used to compare the efficiency of the selected Asian countries in regulating adolescent Corona infection. The output indicator in the present paper is the *reciprocal of the adolescent Corona infection rate.* The output is measured on the vertical axis of Fig. 1. The horizontal axis measures the appropriate societal inputs set.

Given the set of inputs and state of technology, the maximum attainable output is presented by the *frontierr*^35^, and it is presented by the upper line in Fig. 1. Contrarily, the lower line in the figure is the achieved health outcome in the absence of any health facilities. The prime difference between health outcome and firm output is that in the absence of input, output will surely be zero; conversely, health outcome will be a non-zero minimal amount in the absence of health expenditures, simply because all citizens of a country will not die together. Figure 1 presents both the maximum possible as well as the country’s attainable health output, and they are (*e* + *f* + *g*) units and (*e* + *f*) units, respectively. Under this diegesis, *system performance* is the ratio of achieved to potential output^36,37^ that is,

1 $$System\;performance = \frac{f}{f + g}$$

where *f* is the achieved and (*f* + *g*) is the potential output.

Equation 1 is interpreted as "*system achieved compared to its potential*" after Murray, and Frenk^32^. Notably, "*Farrell’s*^35^*, output-based measure of technical efficiency*"^38^ under the present framework allows us to conduct inter as well as intra country health performance comparisons of efficiency in regulating adolescent Corona infection. *Farrell’s*^35^*, measure of technical efficiency* is the ratio of observed to maximum attainable output. Based on SFA, the measurement of the technical efficiency of a country to regulate adolescent Corona infection is facilitated in the paper. The basic model used to explore the said objectives empirically is Battese, and Coelli^39^ *technical inefficiency effects model*. The said model allows us to recognise the output-input relationship and the determinants of a country’s inefficiency to regulate adolescent Corona infection simultaneously.

### Data

This investigation solely relies on secondary data that has been assembled from several secondary sources. The study collected information from 21 Asian nations for the years 2020, 2021, and 2022. Afghanistan, Bangladesh, Cambodia, China, India, Indonesia, Iran, Israel, Japan, Jordan, Kazakhstan, Kyrgyzstan, Lebanon, Malaysia, Nepal, Pakistan, Philippines, Sri Lanka, Thailand, Turkey, and Vietnam are on the list of Asian nations that are covered. The selection of countries as well as data periods is strictly subject to data availability. In this investigation, the data for the main concerned variable, that is, Coronavirus infection among adolescents, and other variables such as hospital beds per thousand and Stringency index are compiled from https://ourworldindata.org/covid-vaccinations. In contrast, data from the World Bank is gathered on per-capita GDP, doctors per 1000 people, nurses and midwives per 1,000 people, population density (people per square km of land area), Internet use (%), urbanisation (%), and regular paid employment (%). Data for Female Political Participation (% in lower parliament) and Tobacco use in adolescents (%) are compiled from http://archive.ipu.org/wmn-e/classif.htm#1 and the World Health Organisation (WHO), respectively. Table 1 includes a detailed description of the variables, their definitions, and their sources:

**Table 1:** Overview of variables.

Variable name	Variable description	Variable category	Data source
Number of adolescent coronavirus infected (*Y*)	Refers to the number of persons infected from COVID-19 among adolescent	Output	https://ourworldindata.org/covid-vaccinations
$\frac{1}{Y}$	Reciprocal of the output variable	–	–
$\ln \left( \frac{1}{Y} \right)$	Logarithm of the output variable. We consider this as our output with the idea lower number of persons infected from COVID-19 among adolescent greater the efficiency	Output	–
Fully vaccinated (%) (Vaccinated)	Shows percentage of population received all vaccination for COVID-19	Input	World Health Organisation
Hospital beds per thousand (Beds)	Shows number of hospital beds per 1000 population	Input	https://ourworldindata.org/covid-vaccinations
Per-capita GDP (PGDP)	GDP per capita is gross domestic product divided by midyear population. Data are in constant 2015 U.S. dollars	Input	World Bank
Doctor per 1000 (Doctors)	Refers to the number of doctors available for per 1000 population on an average	Input	World Bank
Nurses and Midwives per 1000 (Nurses)	Refers to the number of Nurses and Midwives available for per 1000 population on an average	Input	World Bank
Population density (people per sq. km of land area) (Population density)	Number of population lives in per square km of land area	Input	World Bank
Female Political Participation (% in lower parliament) (FPP)	Shows proportion of women members in the lower parliament	Exogenous	http://archive.ipu.org/wmn-e/classif.htm#1
Stringency index (SI)	Measures govt. response towards COVID-19 infection	Exogenous	https://ourworldindata.org/covid-vaccinations
Internet use (%) (Internet)	Displays proportion of population using internet facilities	Exogenous	World Bank
Urbanisation (%) (Urban)	Refers to the segment of population resides in urban areas	Exogenous	World Bank
Tobacco use in adolescents (%) (Tobacco)	Indicates the proportion of adolescent consume tobacco	Exogenous	World Health Organisation
Adolescent suicidal mortality rate (per 100,000)			
Regular wage employment (%) (Employment)	Refers to share of workforce having regular wage and employment	Exogenous	World Bank

*Source*: Authors’ own specification.

### Variables

The list and classifications of the variables utilised in this study are highlighted in this section. We used Stochastic Frontier Analysis (SFA) since our main goal is to examine country-wide efficacy in controlling the severe course of coronavirus among adolescents. However, three sets of variables- output, input, and exogenous are indispensable for establishing input–output orientation using SFA. Below are the specific descriptions of these variables:

#### Output variable

According to the WHO, the term adolescent is defined as a group of people whose age is between 10 to 19 years. It is a special stage of human life for building a concrete foundation for excellent health. Further, adolescents are the key contributors to the future workforce of any country^40^. Therefore, healthy adolescents not only help in human capital formation but also reduce the burden of health expenditure. In the end, this serves as fuel for a nation's economic development. Considering the significance of adolescent health, the present study considers Coronavirus infection among adolescents as an output variable. Although the death rate of adolescents due to the virus, mental health, depression, etc., can reflect the health status of this group during the Corona pandemic, we emphasised only the infection rate based on the availability of data. The decision regarding the output variable is strictly based on data availability. Here, "*reciprocal of coronavirus infection among adolescents*" is selected as our outcome variable because our primary objective is to investigate the efficiency of Asian nations in regulating coronavirus infection among adolescents. We thought about *reciprocal* with the concept that the lower the value, the more efficient it will be in controlling adolescent coronavirus infection. We can take into account the "death rate of adolescents from coronavirus" in particular. A zero value in this situation might lead to inconsistencies in later empirical research; hence, the relevant nation should be removed from the analysis. Furthermore, adolescents are not particularly vulnerable to coronavirus; therefore, a fatality rate of nil is extremely typical. We select "*reciprocal of the coronavirus infection among adolescents*" as our outcome variable in light of this restriction.

#### Input variables

A precise array of inputs is essential for the assessment of the Stochastic Production Frontier (SFA), akin to the output variables. The input variables that represent a nation's health infrastructure and wellness must be included when our aim is to assess the efficiency of health outcomes. Consequently, we have included an assortment of factors that depict a country's health infrastructure, such as the percentage of the population that has received all recommended vaccinations (Fully vaccinated (%)), the number of hospital beds per thousand people, the per-capita GDP, the number of doctors, nurses, and midwives per thousand people, and the population density (the number of people per square kilometre of land), among others. These factors are regarded as inputs because they have a direct or indirect impact on a country's health scenario and outcome. Moreover, the variables hospital beds per thousand, doctors per thousand, and nurses and midwives (per 1000) serve as direct indications of the scenario of the health infrastructure. Furthermore, the country's economic health and population and demographic features are represented by per-capita GDP and population density, respectively, which indirectly affect health outcomes. A higher GDP per capita increases resource availability, which increases the likelihood that health expenditures may increase. Contrarily, rapid illness dissemination, especially infectious diseases, makes epidemics more likely in areas with increased population density^31,41^. The input variables can be measured in a variety of ways, including in monetary form, percentage form, physical form, etc. It is quite challenging to find data in a comparable style because the study contains information pertaining to many Asian countries. Consequently, we incorporate both physical and monetary input variables. Simultaneously, some input variables are expressed in percentage form as well. Table 1 lists the descriptions of the variables.

#### Exogenous variables

It is a fundamental tenet that better health outcomes result from non-health factors as well as greater access to healthcare services and infrastructure^38^. This is because health factors are not sufficient to achieve a positive health outcome. Positive health outcomes require precise socially and economically directed conditions and programmes along with health services or infrastructure^42,43^. Accordingly, in this study, we used a number of non-health factors that are indirectly related to preventing Coronavirus infection in adolescents, including Female Political Participation (% in lower parliament), Stringency index, Internet use (%), Urbanisation (%), Tobacco use in adolescents (%), and Regular wage employment (%). All these factors are termed exogenous variables (the details are presented in Table 1). In this instance, the variables Female Political Participation (% in lower parliament) and Stringency index are used to quantify the effect of female leaders on political decision-making and the government's response to controlling the pandemic scenario, respectively. Simultaneously, urbanisation is used to analyse the aftermath of industrialization and corresponding rural–urban migration on the spread of coronavirus among adolescents in a country. Internet usage serves as a proxy for the level of digitalization and information availability connected to the coronavirus epidemic and its consequent circumstances. Tobacco use in adolescents (%), and Regular wage employment (%) are two exogenous variables that are used in the study to demonstrate negative health behaviours and sustained employment conditions, respectively. These variables also show the effects of these exogenous variables on the spread of the Coronavirus among adolescents.

### Econometric model

The efficiency estimates in this paper will be facilitated by the application SFA, and accordingly, we will estimate SPF. The corresponding SPF for panel specification is given by equation (1):

2 $$y_{it} = g\left( {x_{it} ,\beta } \right)\exp (V_{it} )TE_{it}$$

Here *y* presents health output, *x* and $\beta$ are vectors of arguments and the corresponding vector of parameters to be estimated of the production frontier. *x* includes a number of indicators representing *access and availability* of health infrastructure resources which have direct aftermath on health outcome. All variables are transformed into logarithm form to take care of the *heteroscedasticity* problem. $\exp (V_{it} )$ presents the random error component which is outside the control of the country. The equation includes another error component $TE_{it}$ and it is expressed as $TE_{it} = \exp \left( { - U_{it} } \right)$ after Kumbhakar^44^. The assumption related to $TE_{it}$ is $TE_{i} \le 1$ hence $U_{i} \ge 0$^44^. Thus the error is one-sided. Equation (2) in modified form may be written as:

3 $$y_{it} = g\left( {x_{it} ,\beta } \right)\exp (V_{it} )\exp \left( { - U_{it} } \right)$$

The assumptions concerning the two error terms are:

$$V_{it} \sim IIDN\left( {0,\sigma_{V}^{2} } \right)\;{\text{and}}\;U_{it} \sim IIDN\left( {\omega_{it} \theta ,\sigma_{U}^{2} } \right).$$

Here, $\omega_{it}$ is a (*pxq*) vector of exogenous variables which are supposed to influence the controllable inefficiency of the country related to health outcome. The corresponding unknown parameters are presented by $\theta$, a vector of (*qxp*).

The corresponding distribution of both error terms (random and technical efficiency) are Normal-Truncated Normal as directed in Stevenson^45^. Notably, both the error terms are independent of each other and also independent of the explanatory variables. After Battese and Coelli^39^, the identification of the determinants of technical efficiency is facilitated by the following equation:

4 $$TE_{it} = \exp ( - U_{it} ) = \exp ( - \omega_{it} \theta - \varepsilon_{it} )$$

$\varepsilon_{it}$, the random variable follow truncated normal distribution with ‘0’ mean and $\sigma_{U}^{2}$ variance. Here, $\varepsilon_{it} \ge - \omega_{it} \theta$, implying point of truncation is $- \omega_{it} \theta$. The maximum likelihood estimators of the parameters of both stochastic production frontier and the inefficiency effects model can be obtained simultaneously by using FRONTIER-4.1^39,46^. The corresponding variance parameters are:

$\sigma^{2} = \sigma_{V}^{2} + \sigma_{U}^{2}$ and $\gamma = \frac{{\sigma_{U}^{2} }}{{\sigma^{2} }}$, where $\gamma$ ranges from 0 to 1 based on the dominant force of $\sigma$ and $\sigma_{u}$ respectively.

Based on our specified list of output, inputs and exogenous variables, the Battese and Coelli^39^ assuming a log-linear version of the Cobb–Douglas model can be expressed as follows:

5 $$\begin{aligned} & \ln \left( {\frac{1}{{Y_{it} }}} \right) = \alpha_{0} + \alpha_{Vaccinated} \ln (Vaccinated) + \alpha_{Beds} \ln (Beds) + \alpha_{PGDP} \ln (PGDP) + \alpha_{Doctors} \ln (Doctors) \\ & \quad + \alpha_{Nurses} \ln (Nurses) + \alpha_{Population\;density} \ln (Population\;density) + \left( {V_{it} - U_{it} } \right) \\ \end{aligned}$$

Here, *ln* presents natural logarithm.

The corresponding inefficiency effects equation is present as follows:

6 $$\begin{aligned} & U_{it} = \theta_{0} + \theta_{WPP} \ln (FPP) + \theta_{SI} \ln (SI) + \theta_{Internet} \ln (Internet) + \theta_{Urban} \ln (Urban) \\ & \quad + \theta_{Tobacco} \ln (Tobacco) + \theta_{Employment} \ln (Employment) + \varepsilon_{it} \\ \end{aligned}$$

Here, $U_{it}$ is *ith* country’s controllable inefficiency for *tth* time period and *FPP* is the Female Political Participation (% in lower parliament), *SI* is Stringency index, *Internet* is Internet use (%), *Urban* is Urbanisation (%), *Tobacco* is Tobacco use in adolescents (%) and *Employment* presents Regular wage employment (%).

The application of the statistical packages FRONTIER-4.1^46^ gives maximum likelihood estimates of parameters of (5) and (6) simultaneously.

## Results

The empirical results of this pre-dominantly empirical study is presented in this section.

### Adolescent behavioural changes in selected Asian countries in pre-and-post pandemic scenario

**Figure 2:**
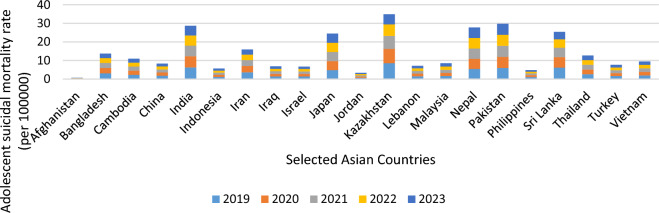
Adolescent suicidal mortality rate (per 100,000) across Asian Countries. Source: Authors’ own presentation based on World Bank data.

**Figure 3:**
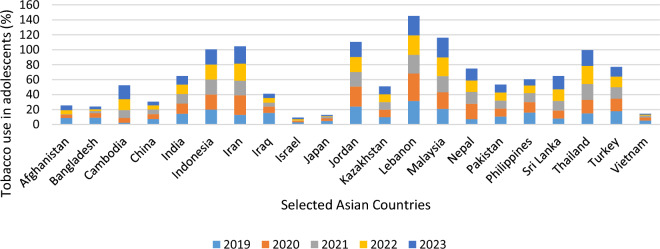
Tobacco use in adolescents (%) across Asian countries. Source: Authors’ own presentation based on World Bank data.

The adolescents’ behavioural changes in the current paper are analysed based on two indicators, viz., adolescent suicidal mortality rate (per 100,000) and tobacco use in adolescents (%) from 2019 to 2023. The choice of the variables and the study period for this purpose are completely dictated by the availability of the data. For the entire study period, Kazakhstan reports the highest mean for "adolescent suicidal mortality rate (per 100,000)", followed by India, and the lowest corresponding figure is obtained for Afghanistan. Contrarily, considering tobacco use in adolescents (%), the highest figure is obtained for Lebanon for the entire study period, and the corresponding lowest figure is reported for Cambodia in 2019 and Israel for the subsequent study period. Thus, we can say that there is not much variation in the adolescents’ behaviour considering these two indicators before and after the pandemic. The information is presented graphically in Figs. 2 and 3, respectively.

The summary statistics of these two variables for selected Asian countries are presented in Table 2.

**Table 2:** Summary statistics of adolescent behavioural changes in selected Asian countries.

Adolescent suicidal mortality rate (per 100,000)					Tobacco use in adolescents (%)			
Country	Mean	SD	Minimum	Maximum	Mean	SD	Minimum	Maximum
Afghanistan	0.14	0.05	0.08	0.2	5.10	3.02	0.7	8.9
Bangladesh	2.74	0.26	2.41	3.06	4.80	2.83	2.4	9.2
Cambodia	2.19	0.10	2.06	2.31	10.50	6.40	2.4	18.6
China	1.66	0.12	1.52	1.81	6.10	0.95	4.90	7.3
India	5.74	0.41	5.22	6.26	13.00	1.03	11.7	14.3
Indonesia	1.13	0.02	1.11	1.16	20.10	0.08	20	20.2
Iran	3.18	0.34	2.75	3.61	20.90	5.06	12.9	26.2
Israel	1.36	0.06	1.29	1.44	8.24	4.35	5.1	15.7
Japan	1.33	0.08	1.24	1.43	1.86	0.93	0.5	3.1
Jordan	4.89	0.07	4.81	4.98	2.56	1.59	0.9	4.8
Kazakhstan	0.67	0.01	0.66	0.68	22.07	3.18	19.5	26.75
Kyrgyzstan	6.97	1.22	5.43	8.51	10.20	0.32	9.8	10.6
Lebanon	1.42	0.05	1.36	1.48	29.05	4.95	25	36.65
Malaysia	1.70	0.06	1.63	1.77	23.20	2.31	20.9	26.3
Nepal	5.55	0.10	5.42	5.68	14.96	4.82	7.2	20.5
Pakistan	5.95	0.01	5.94	5.96	10.70	0.00	10.7	10.7
Philippines	0.96	0.08	0.86	1.07	12.10	3.08	8.20	16
Sri Lanka	5.08	0.86	4.00	6.16	13.00	3.87	8.1	17.9
Thailand	2.53	0.04	2.48	2.59	19.90	3.49	15	24.15
Turkey	1.53	0.08	1.43	1.63	15.40	1.98	12.9	17.9
Vietnam	1.89	0.05	1.82	1.96	2.90	1.80	1.2	5.4

*Source*: Authors’ own calculation based on World Bank data.

Table 2 discloses that the highest mean of the variable, "adolescent suicidal mortality rate (per 100,000)" is reported for Kyrgyzstan (6.97), and the country is followed by Pakistan (5.95). Notably, Pakistan reports the lowest SD (0.01), while Kyrgyzstan (1.22) reports the corresponding highest figure. This means that although the adolescent suicidal mortality rate (per 100,000) in Kyrgyzstan is high, the highest SD indicates it will not persist in the future; however, the same conclusion is not true for Pakistan. The high value of SD indicates that the higher mean value is also likely to persist in the future.

The highest mean value of tobacco use in adolescents (%) is obtained in Lebanon (29.05), followed by Malaysia (23.2). The corresponding SD value for Cambodia is also high (4.95). This indicates the tendency towards higher tobacco consumption among adolescents’ is likely to persist in Cambodia in the near future. Contrarily, Japan reports the lowest mean, 1.86, in this regard, and the corresponding SD is also very low, 0.93. This indicates Japan will be successful in maintaining a lower tobacco addiction among adolescents in the near future also.

### Two-way ANOVA of adolescent behavioural changes in selected Asian countries

The discussion above makes it clear that no appreciable behavioural changes were noticed in Asian adolescents’ population before or during the epidemic. However, it is noteworthy that adolescent behaviour varies among Asian nations. Only a graph or the analysis of descriptive statistics may not be able to clearly show such a discrepancy. Conducting an ANOVA test is the best technique to identify differences in adolescent behaviour among Asian nations. A two-way non-parametric ANOVA will be appropriate for this purpose because we are dealing with panel data. This study used a two-way non-parametric ANOVA test, and the results are shown in Table 3 for both indicators.

**Table 3:** Two-way ANOVA of adolescent behavioural changes in selected Asian countries.

Adolescent suicidal mortality rate (per 100,000)							Tobacco use in adolescents (%)					
Source of Variation	SS	df	MS	F	*P*-value	F crit	SS	df	MS	F	*P*-value	F crit
Nation wise	1289.82	44	29.31	259.62	0.00	1.45	11,574.37	44	263.05	19.54	0.00	1.45
Year wise	1.38	4	0.35	3.06	0.02	2.42	136.46	4	34.11	2.53	0.04	2.42
Error	19.87	176	0.11			2369.34	176	13.46				
Total	1311.08	224				14,080.17	224					

*Source*: Authors’ own calculation based on World Bank data.

The table reveals that there are two comparable F-statistics to represent differences in the two specified variables by country and by year. Notably, both the F-statistics, which are, country- and year-wise, are significant at the 1% level for the two variables, viz., adolescent suicidal mortality rate (per 100,000) and tobacco usage in adolescents (%). The associated F-statistics value is excessive in each of the four situations. This enables us to rule out the null hypothesis that there is no regional variation, and we draw the conclusion that adolescents conduct varies significantly among Asian nations. These behavioural variations may affect how Asian adolescents' Corona infection is regulated.

### Disparities in adolescent corona infection of selected Asian countries

The outbreak of Corona has several consequences for human life. Every age group on the planet, including adolescents, is impacted. Although adolescents seem to be at relatively low risk for the virus, it substantially disrupts their lives in a number of ways. Health issues, social exclusion, and school closures have an impact on people's physical and mental health as well as their social lives. Research focused on the insinuations of Corona suggests that the level of anxiety, depression, and substance abuse among the adolescent population has significantly increased around the globe during the Corona pandemic^16,17^. Several current studies indicate that the fear of the virus, social isolation, and the demands of the new educational model are some of the key factors contributing to this difficult situation^18^.

**Figure 4:**
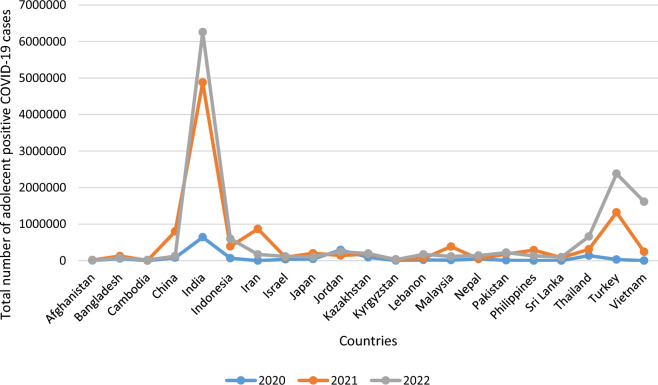
Total number of adolescents' Coronavirus infection across selected Asian countries. Source: Authors’ own presentation based on World Bank data.

Despite the fact that there has been less Corona contamination among adolescents, WHO, ^6^ states that this may be because there hasn't been enough testing and there aren't any overt signs of infection among the aforementioned demographic. It is undeniably true, nevertheless, that the infection may raise the likelihood of long-term health issues. Figure 4 shows disparities in adolescent Corona infection among selected Asian nations.

**Figure 5:**
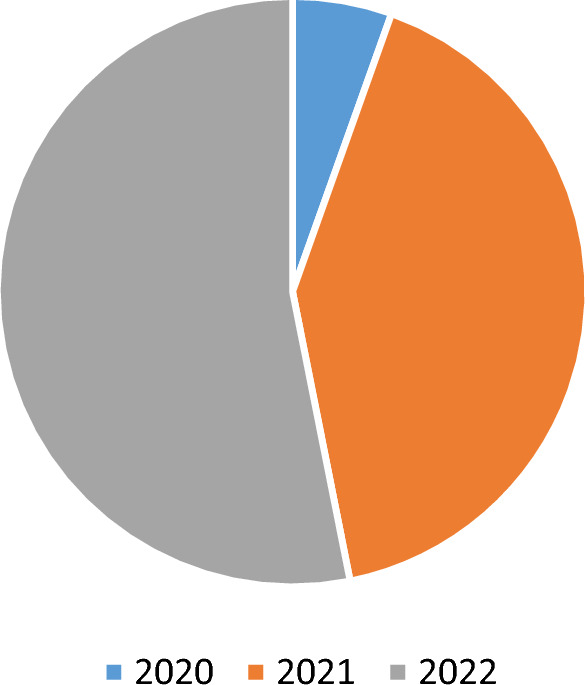
Pie-chart of total number of adolescents' Coronavirus infection in India over time. Source: Authors’ own presentation based on World Bank data.

The aforementioned graph demonstrates the stark differences in the spread of Coronavirus infection among adolescents in the selected Asian nations. From 2020 to 2022, India was one of these countries that seemed to be most impacted. In fact, as seen in Fig. 5, India had an increase in the number of infected adolescents during the time period.

Following India, Jordan, Thailand, Kazakhstan, and China are the most severely affected nations concerning adolescent Corona confirmed cases during the period. Interestingly, it is also seen from Fig. 4 that, although China is the first country where the Coronavirus has been detected and has endured many condemnations, it is effectively able to arrest the virus spread among adolescents in 2020 to a greater extent.

The discrepancies in regulating adolescents’ coronavirus infection may also be noticed from the summary statistics as presented in Table 4.

**Table 4:** Summary statistics of adolescent Corona infection in selected Asian countries.

Country	Mean	SD	Minimum	Maximum
Afghanistan	10,251.23	5706.025	5134.4	16,404.4
Bangladesh	85,152.97	36,357.37	56,151.1	125,941.3
Cambodia	8647.567	9846.626	49.1	19,389.3
China	334,685.8	403,341.3	81,645.3	799,824
India	3,924,522	2,925,706	641,160	6,254,974
Indonesia	352,604.2	268,899.2	67,258	601,295.8
Iran	346,165.8	458,950.9	894.3	866,977.8
Israel	82,000.43	40,981.49	36,528.4	116,081.2
Japan	117,495.1	78,749.59	46,060.8	201,939.6
Jordan	226,413.8	77,352.84	141,594.7	293,067
Kazakhstan	156,977.3	56,127.82	92,718	196,413.8
Kyrgyzstan	22,549.03	16,666.62	3636.8	35,090.91
Lebanon	82,982.18	78,805.97	19,464.7	171,172.5
Malaysia	172,560.4	191,677.8	15,467.9	386,132
Nepal	78,182.87	53,663.1	46,359.3	140,140
Pakistan	137,168.1	112,184.3	9632.6	220,591.1
Philippines	140,208.5	143,468	1602	288,088.2
Sri Lanka	58,791.62	51,175.32	96.8	94,063.76
Thailand	370,465.5	266,675.8	138,354	661,761.5
Turkey	1,244,323	1,177,011	30,466	2,380,655
Vietnam	618,700	870,007	203.84	1,613,520

*Source*: Authors’ own calculation based on World Bank data.

Table 4 discloses that the highest average infection rate among adolescents’ is obtained for India (3,924,522) and the corresponding lowest figure is for Cambodia (8647.567). The maximum dispersion in regulating adolescents’ coronavirus infection, as dictated by SD, is obtained again for India, and the lowest figure is reported for Afghanistan. This indicates that India's success in lowering coronavirus infection among adolescents will only be fleeting.

Discrepancies in countries performances encourage us to explore the efficacy of Asian countries in regulating adolescent Corona spread. However, a country's efficiency in controlling adolescents’ Corona cannot be understood solely by diagrammatic analysis. To understand a country's efficacy in controlling severe infections among adolescents, an in-depth assessment is needed. Prior to that, it's crucial to examine regional variations in regulating adolescents’ coronavirus infection.

### Two-way ANOVA of adolescent Corona infection of selected Asian countries

After scrutinising the nation-wide scenario of coronavirus infection among adolescents in Asia, we noted that there is considerable variation in the extent of infection. However, it is not possible to establish the fact only by considering a diagrammatic explanation. In this regard, ANOVA is one of the important techniques that helps to examine whether there is any significant variation or not. Particularly in this paper, we have employed a two-way ANOVA to establish if there is any significant variation in coronavirus infection among adolescents or not. The result of the ANOVA is displayed in Table 5:

**Table 5:** Two-way ANOVA of adolescent Corona infection in selected Asian countries.

Source of variation	*SS*	*df*	*MS*	*F*	*P*-value	*F crit*
Nation wise	4.36E + 13	20	2.18E + 12	4.592998	0	1.838859
Year wise	3.67E + 12	2	1.84E + 12	3.874155	0.028976	3.231727
Error	1.9E + 13	40	4.74E + 11			
Total	6.62E + 13	62				

*Source*: Authors’ own calculation based on World Bank data.

The result of the ANOVA visualised in Table 5 provides two different types of F statistics. The F statistic concerning nation-wise variation suggests that there is significant variation in the extent of Corona infection among adolescents in selected Asian countries. This is true because the F statistic (4.59) with 20 degrees of freedom is statistically meaningful with less than 1% significance. Again, the significant value of the F statistic (3.87) concerning year-wise variation also confirms the existence of significant variation in the extent of infection during the periods. Therefore, the result of the ANOVA confirms that the extent of infection varies not only across years but also across selected Asian nations. In this light, it will be appropriate to examine the efficiency of different Asian countries in regulating adolescents’ coronavirus infection.

### Pesaran's test of cross sectional independence

A common phenomenon of panel data is that it exhibits *erroneous cross-sectional dependency in error*^47^. Such inter-correlation not only makes the estimators of either fixed effects or random effects models unbiased but also inconsistent. Thus, prior to estimating any model of panel data it is necessary to check *cross-sectional independence* of the error. In this regard, the Pesaran test^48^, is the most acclaimed method. The test result is presented in Table 6.

**Table 6:** Pesaran's test of cross sectional independence.

Pesaran’s test of cross sectional independence	Average absolute value of the off-diagonal elements	Probability
−1.252	0.651	1.7893*

*Source*: Authors’ own calculation based on secondary data.

*Evidence shows data are cross-sectionally independent.

The test result concludes no cross-sectional dependence, as the corresponding "*Pesaran’s test of cross-sectional independence*" value is −1.252 with a probability of 1.7893. Moreover, the value of the "*average absolute value of the off-diagonal elements*" is 0.651. Thus, we accept the null hypothesis of no cross-sectional correlation in errors.

### Factors influencing adolescent Corona infection of selected Asian countries

We used the panel regression approach to examine the key factors of Coronavirus infection in adolescents, as our inquiry is based on panel data. Fixed Effects (FE) and Random Effects (RE) regression are two distinct types of regression results that are produced using panel regression analysis. The findings are shown in Table 7.

**Table 7:** Random-effects GLS regression and Fixed-effects (within) regression of stochastic production frontier function for whole panel.

Variable	Random effects				Fixed effects			
	Coefficients	SE	t-ratio	${\text{P}} > \left| {\text{t}} \right|$	Coefficients	SE	t-ratio	${\text{P}} > \left| {\text{t}} \right|$
Constant	0.018	0.012	1.49	0.135	−48.827	50.805	−0.96	0.344
Fully vaccinated (%)	−0.082	0.013	−6.12	0	−0.105	0.043	−2.44	0.021
Hospital beds per thousand	−0.002	0.244	−0.01	0.993	16.455	12.608	1.31	0.201
Per-capita GDP	0.000	0.000	−0.41	0.683	−0.001	0.000	−1.56	0.128
Doctor per 1000	−1.793	0.788	−2.28	0.023	−4.706	2.746	−1.71	0.097
Nurses and Midwives (per 1000)	0.050	0.204	0.24	0.809	0.297	0.281	1.06	0.298
Population density (people per sq. km of land area)	0.000	0.002	0.04	0.971	0.098	0.069	1.42	0.164
Female Political Participation (% in lower parliament)	0.038	0.069	0.56	0.577	0.000 (omitted)			
Stringency index	−9.323	2.644	−3.53	0	0.045	0.031	1.46	0.153
Internet use (%)	0.049	0.036	1.35	0.176	0.027	0.095	0.29	0.776
Urbanisation (%)	−0.029	0.031	−0.94	0.348	−0.110	0.830	−0.13	0.896
Tobacco use in adolescents (%)	−0.084	0.071	−1.19	0.235	−0.334	3.839	−0.09	0.931
Regular wage employment (%)	0.061	0.039	1.56	0.119	−0.145	0.414	−0.35	0.729
$R^{2}$*(Within)*	0.624				0.697			
$R^{2}$*(Between)*	0.307				0.0004			
$R^{2}$*(Overall)*	0.448				0.0000			
Wald chi2(12)	62.72				6.49			
Prob > chi2	0.000				0.000			
*Sigma_u*	1.455				46.867			
*Sigma_e*	1.326				1.326			
*Rho*	0.546				0.999			

*Source*: Authors’ own calculation based on secondary data.

***, **, * significance at 1%, 5% and 10% respectively.

We begin our investigation with the RE model. The findings demonstrate that Full vaccination, Doctor per 1000, and Stringency index all significantly affect the control of adolescent Corona infections. Among these factors, the negative value of the estimated coefficients of full vaccination suggests that the higher the level of fully vaccinated adolescents, the lower the likelihood of infection among them. Again, negative doctor per 1000 coefficients suggest that having more physicians around will assist to reduce infection. As evaluated by the Stringency Index, the model also predicts that a stronger government response to the virus will help reduce infection in the chosen nations. Contrarily, the results of the FEM show that the only important influences on adolescents’ infection are full vaccination and the doctor per 1000 variables. Similar to REF, FEM shows that full vaccination and the number of doctors per 1000 patients have a detrimental effect on adolescents’ infections. This suggests that increasing the use of these predictors might lower the risk of infection among adolescents in the Asian nation. The discussion section talks about the possible reasons for these empirical findings.

We may assume that REM is more appropriate than FEM based on the values $R^{2}$ (Within), $R^{2}$ (Between), and $R^{2}$ (Overall) for goodness of fit. Nevertheless, selecting the best model is not reasonable based solely on the quality of fit. In this case, the Hausman test is required to confirm that the particular panel regression model is adequate and suitable. The subsequent section analyses the outcome of the Hausman test.

### Hausman test and model selection

For panel regression analysis, there are two different types of effects models: Fixed Effects Model and Random Effects Model. We may compare the two models using the Hausman test for specification, which also helps us select the best model for analysis. Table 8 presents an overview of the test's outcomes.

**Table 8:** Hausman Test to choose between Random Effects and Fixed Effects Model.

Variables	Coefficients		Difference (b-B)	$\chi_{10}^{2}$	**Prob **$> \chi^{2}$
	Fixed effects	Random effects			
Fully vaccinated (%) (Vaccinated)	−0.1054	−0.0821	−0.0232	15.24	0.1234
Hospital beds per thousand (Beds)	16.4550	−0.0022	16.4572		
Per-capita GDP (PGDP)	−0.0008	0.0000	−0.0007		
Doctor per 1000 (Doctors)	−4.7057	−1.7932	−2.9125		
Nurses and Midwives per 1000 (Nurses)	0.2967	0.0495	0.2472		
Population density	0.0981	0.0001	0.0981		
Stringency index (SI)	0.0451	0.0179	0.0273		
Internet use (%) (Internet)	0.0273	0.0487	−0.0214		
Urbanisation (%) (Urban)	−0.1095	−0.0289	−0.0806		
Tobacco use in adolescents (%) (Tobacco)	−0.3344	−0.0840	−0.2504		
Regular wage emp. (%) (Employment)	−0.1445	0.0612	−0.2057		

*Source*: Authors’ own calculation based on secondary data.

*Evidence shows Random Effects model is appropriate.

The results of the Hausman test presented in Table 8 suggest that the value of $\chi_{10}^{2}$ is 15.24 and it is not statistically meaningful as its probability is, *Prob* $> \chi^{2}$** = **0.123. Therefore, the test results authenticate that the RE model is suitable for this inquiry. This again authorises us to apply the technical inefficiency effects model in the SFA framework. Consequently, we have utilised the technical inefficiency effects model in the stochastic production function related to panel data^49^.

### Efficiency estimates across Asian countries in regulating adolescent Corona

The efficiency estimates of different Asian countries in regulating adolescent Corona infection are discussed in this section. The divergences in the data are ensured by the summary statistics of the involved regression variables (see Table A.1). The efficiency score of all the Asian countries in regulating Corona is presented in Table 9. The benchmark for separating efficient countries from inefficient ones is the overall panel mean efficiency score of 0.3718^50^. Correspondingly, if a country’s efficiency score exceeds the overall panel mean efficiency score, the country will be recognised as technically efficient; otherwise, it will be technically inefficient. In particular, the efficiency score of Malaysia is 0.7263, greater than the overall mean efficiency score of 0.3718, and thus the country is considered to be technically efficient in regulating adolescent Corona infection.

**Table 9:** Efficiency score of regulating adolescent Corona infection of Asian countries.

Country	2020	2021	2022	Panel mean efficiency	Ranking
**Afghanistan**	**0.0006**	**0.0005**	**0.0003**	**0.0004**	**21**
Bangladesh	0.0232	0.0286	0.0056	0.0191	18
Cambodia	0.7920	0.7561	0.1360	0.5614	9
China	0.4957	0.5944	0.7623	0.6175	7
**India**	**0.0048**	**0.0089**	**0.0032**	**0.0056**	**20**
Indonesia	0.0691	0.0813	0.0322	0.0608	14
Iran	0.1067	0.1051	0.0644	0.0921	13
**Israel**	**0.9236**	**0.9301**	**0.7748**	**0.8761**	**2**
Japan	0.3050	0.3979	0.4531	0.3853	10
Jordan	0.8642	0.8697	0.2362	0.6567	6
**Kazakhstan**	**0.9538**	**1.0000**	**0.9131**	**0.9557**	**1**
**Kyrgyzstan**	**0.8676**	**0.8755**	**0.7546**	**0.8326**	**3**
Lebanon	0.0699	0.0887	0.0215	0.0600	**16**
Malaysia	0.8781	0.8969	0.4039	0.7263	4
Nepal	0.0357	0.0620	0.0404	0.0460	**17**
**Pakistan**	**0.0155**	**0.0148**	**0.0099**	**0.0134**	**19**
Philippines	0.8375	0.8190	0.3700	0.6755	5
Sri Lanka	0.6914	0.7700	0.2728	0.5781	8
Thailand	0.0534	0.0957	0.0311	0.0600	15
Turkey	0.3656	0.4364	0.0908	0.2976	11
Vietnam	0.4220	0.3422	0.0978	0.2873	12
Mean Efficiency (Yearly)	**0.4179**	**0.4368**	**0.2607**	**−**	
Mean Efficiency (Overall)	**0.3718**				

*Source*: Authors’ own calculation based on secondary data.

Bold values are indicatng the highest and lowest values in the list.

Based on this benchmark, 10 out of 21 countries have become efficient in regulating adolescent CORONA infection. This indicates that, on average, 48% of countries are efficient in regulating adolescent CORONA infection. Furthermore, based on this benchmark, the highest efficiency is obtained for Kazakhstan (0.9557), followed by Israel (0.8761) and Kyrgyzstan (0.8326). Contrarily, the least efficient country on the list is Afghanistan (0.0004), followed by India (0.0056) and Pakistan (0.0134). As the analysis involved panel data, we have also performed a year-wise comparison of efficiency. Considering yearly mean efficiency as a benchmark, we observe that in 2020, 2021, and 2022, only 10, 9, and 8 countries, respectively, out of 21 are efficient in regulating adolescent Corona infection. This indicates, based on yearly mean efficiency scores of 48%, 43%, and 38%, that countries are efficient in regulating adolescent CORONA infection in 2020, 2021, and 2022, respectively. The best and worst performers on the list are Kazakhstan and Afghanistan, respectively, in 2020, and the ranks remain unaltered in the subsequent years as well.

Notably, the efficiency score is a relative performance score, and it is only an indicator of how well one country is performing relative to the other in regulating adolescent CORONA infection. This efficiency score *does not specify a hierarchy in relation to real health outcomes*. In particular, the panel mean efficiency score of Bangladesh is only 0.0191 and holds the 18^th^ rank in the list. The country is recognised as technically inefficient based on our benchmark. However, when considering the actual adolescent Corona infection rate, Bangladesh holds the 8^th^ position with a mean adolescent Corona infection of 85,152.97. The efficiency score of the existing health system stipulates that if *Bangladesh* could operate its health system as efficiently as *Kazakhstan*, the country could regulate adolescent Corona infection more appropriately.

### Technical inefficiency effects: determinants of efficiency

The output-input orientation as well as the determinants of country specific inefficiency are discussed in this section. The empirical findings related to technical inefficiency effects model is presented in Table 10.

**Table 10:** Maximum likelihood estimates of the SPF of regulating adolescent Corona infection for selected Asian countries.

Variables			Coefficient	SE		t-ratio
Constant		$\beta_{0}$	0.034	0.291		0.116
Fully vaccinated (%) (Vaccinated)		$\beta_{1}$	1.937***	0.474		4.088
Hospital beds per thousand (Beds)		$\beta_{2}$	–1.051*	0.609		–1.724
Per-capita GDP (PGDP)		$\beta_{3}$	–0.374	0.270		–1.382
Doctor per 1000 (Doctors)		$\beta_{4}$	1.489***	0.696		2.139
Nurses and Midwives per 1000 (Nurses)		$\beta_{5}$	1.067***	0.486		2.197
Population density (people per sq. km of land area) (Population density)		$\beta_{6}$	–2.829*	1.464		–1.932
Female Political Participation (% in lower parliament) (FPP)		$\delta_{1}$	–0.092	0.061		–1.522
Stringency index (SI)		$\delta_{2}$	–0.049***	0.018		–2.632
Internet use (%) (Internet)		$\delta_{3}$	–0.058***	0.026		–2.250
Urbanisation (%) (Urban)		$\delta_{4}$	0.059***	0.024		2.419
Tobacco use in adolescents (%) (Tobacco)		$\delta_{5}$	1.164***	0.436		2.672
Regular wage employment (%) (Employment)		$\delta_{6}$	–0.100***	0.041		–2.410
$\hat{\sigma }_{s}^{2}$			2.569***	0.787		3.266
$\gamma$			0.251***	0.049		5.132
$\mu$			12.315***	2.991		4.118
*Log(likelihood)*			–119.784			
*LR test of one sided error*	25.817		$Prob > \chi^{2}$		0.004	

*Source*: Authors’ own calculation based on secondary data.

***, **, *Significance at 1%, 5% and 10% respectively.

The first part of the analysis involves the discussion of the output-input relationship in the SPF framework. The output variable is the reciprocal of the *adolescent Corona infection*. The analysis involves *six* inputs and *vaccinated, beds, doctors, nurses, and population density* have statistically meaningful influences on output. The sign of the estimated coefficients predicts that an escalation of *vaccinated, doctors, nurses* reduces *adolescent Corona infection*. Contrarily, an increase in *beds and population density* increases *adolescent Corona infection*. The possible explanation of such an empirical result is discussed in the *discussion* section.

Particularly intriguing are the actual findings of the estimated coefficients of the inefficiency impacts coefficients. The negative sign of the computed coefficient predicts that the country's controllable inefficiency in regulating adolescent Corona infection may be diminished by focusing on these elements. The current analysis involves *six exogenous* variables.

The factors that have a substantial impact on the country's inability to reduce the *adolescent Corona infection* are *SI, urban, internet, tobacco and employment*. Here, *SI* is a proxy for good governance. The improvement of *SI, internet and employment* escalates the efficiency of a country to regulating *adolescent Corona infection*. Contrarily, an increase in *adolescent tobacco* consumption and *urbanization* enhances the inefficiency of a country's efforts to regulate *adolescent Corona infection*. All the estimated coefficients are significant at a permissible level. Here, *WPP* is the proxy for women’s political empowerment. Interestingly, the sign of the *WPP* predicts that an enhancement of women’s political empowerment escalates the efficiency of the country in regulating *adolescent Corona infection*. However, because of the time invariances of the data, the estimated coefficient becomes statistically insignificant. The *discussion* section deals with the explanation of such empirical results.

Notably, each of the included variance parameter is significant at various levels. The statistical significance of the variance parameter, $\gamma$ validates the assumption of a *half normal distribution* of the error term. Here, the value of $\hat{\sigma }_{s}^{2}$ is 2.569 which is statistically significant, implies the accuracy of the provided assumptions about the distribution of the composite error term. The value of $\gamma$ is 0.251 and it is statistically significant at the 1% level. This insinuates that country-specific technical efficiency is crucial in explaining the overall variability of the health system. Additionally, the model is viable, as seen by the estimate linked to the sigma-squared of the technical inefficiency effects, which is rather substantial. Indicating that the inefficiency effects are anticipated to be particularly important for Asian countries in lowering the *adolescent Corona infection*, the estimate for the variance parameter,$\gamma$ is considerably different from "0". The variance parameters' statistical significance validates the model and makes it appropriate for usage.

The model convergences is authenticated by the *Log(likelihood)* value of -119.784 and the high value of Likelihood Ratio Chi-squares 25.817 also take the adequacy of the specification into consideration.

## Discussion

The possible explanation of the empirical result is presented in this section. As we have utilised^39^, "technical inefficiency effects" model. Hence, it has two subsequent parts of estimates, viz., the production frontier and the inefficiency parameters. The output-input relation reveals that the regulation of adolescent Coronavirus spread is influenced by *Vaccinated, Beds, Doctors, Nurses and Population density* at various levels of significance. A better adolescent’s vaccination status decreases the risk of contracting Corona, according to the sign of the estimates for the regressor "*Vaccinated*". The patent conclusion is reinstated as a result. Vaccination serves as a preventative measure since it is well acknowledged that "prevention is better than cure^51,52^". The risk that a person would be seriously or even at all afflicted by the virus decreases with vaccination, and this is true for adolescents everywhere^38,53,54^. The spread of Corona in adolescents in Asian nations is accelerated by an increase in hospital beds. Here, hospital beds represented the enormity of the scenario. Generally, hospital beds are increased to accommodate the increasing number of serious patients who cannot be treated at home^55,56^. Consequently, more serious Corona cases are reported when hospital beds are increased^38^. Therefore, a rise in Bends is associated with more severe Corona instances, especially those involving adolescents. Doctors and nurses are seen as health fighters on a global scale. The present paper considers *Doctors and Nurses* as the protectors of all from the Coronavirus. Similar to prior research by Biswas et al.^57^ and Maity and Barlaskar^38^, an increase in their numbers here also lessens the risk that the lethal virus would spread widely among Asian adolescents. Regardless of vaccination rates, the highly contaminated Coronavirus spread quickly in densely populated areas^58,59^. Thus, highly dense populations always act as favourable stimulants for Corona^38,60^. Due to the dense population in Asia, the virus spread like a cyclone even in the face of significant pre-causational precautions like lockdown, staying at home, etc.^61,62^. Adolescent residents in densely populated areas are more likely to contract Corona^62^. The same is also predicted by our empirical findings.

The estimates of inefficiency determinants are fascinating. Our model predicts, following exogenous factors are most effective: *Stringency index (SI), Internet, Urban, Tobacco and Employment*. Notably, the negative sign of the estimates indicates the positive influence of that exogenous factor in elevating the efficacy of a country in regulating adolescent Corona. The present paper includes *Stringency index* as a replica of good governance. Good governance practise helps in getting favourable outcomes in all circumstances^63^. The negative and statistically meaningful estimates predict the same in our case as well. An increase in *SI* indicates an improvement in good governance practises and eventually aids in achieving desired goals, including regulating Corona spread among adolescents. The lack of such research prevents us from presenting a comparable finding. The estimates of two other exogenous factors, *Internet and Employment*, predict that an improved internet and guaranteed employment facilities escalate the efficacy of a country in controlling the spread of Coronavirus among adolescents. Both of these factors increase the likelihood of having a successful *lockdown, and* the well-accepted techniques to stop the Coronavirus are spared^38^. Improved internet facilities, help in work-from-home, online marketing, online-doctor consultation, online-medicine availability, and most importantly, for adolescent online school, colleges, and interaction with friends^64,65^, particularly help in having a semi-normal life in a pandemic scenario. Consequently, improved internet facilities enhance the efficacy of a country in regulating adolescent Coronavirus spread^38^. High school adolescents sometimes engage in part-time jobs. Lockdown creates difficulties for them in continuing their current part-time employment^66^. Those who enjoyed work from home facilities and monetary support from family coped with this situation more easily^67^. Adolescents feel relieved when the primary breadwinners of the family have formal work with guaranteed salaries; as a result, they choose to stay at home during a *lockdown* in order to protect their families as well as themselves^38,67^. The estimates of the factor *Urban* predict that increased urbanisation increases the likelihood of the rapid spread of Corona among Asian adolescents. In most Asian countries, increased urbanisation has resulted in the end of rural-to-urban migration in search of work^68^. These low-wage workers live in substandard conditions with their families on the outskirts of the main metropolis^67^. Due to their subpar living conditions, inappropriate food consumption, improper satiation, and most significantly, insufficient medical services, these individuals are in high danger^68^. They embrace numerous intoxicants, which increases their propensity to contract any illness, including the Coronavirus^69^. Because of this, we observe the negative influence of urbanisation on Corona spread among adolescents^68^. The increased *consumption of tobacco by adolescents* is a global treat^70^. To restrict such consumption around the globe, legal restrictions exist^71^. Adolescents can occasionally develop a tobacco addiction unintentionally^71^. Their internal health state is discreetly and gradually destroyed by tobacco use and addiction^71^. Any virus may readily infect a frail body, and Corona is no different in this regard^72^. This is the reason for the positive significant value of the estimates of the exogenous factor *Tobacco*, which predicts an escalation in adolescent tobacco consumption and increases the inefficiency of the state in regulating adolescent Coronavirus spread. The absence of an efficiency study considering tobacco consumption as an explanatory factor restricts us from reporting any earlier study. Notably, the inclusion of both *Stringency index and Tobacco* stipulates the novelty of the study. Notably, unlike earlier studies, the estimate of *Female Political Participation (FPP)* fails to influence the administration of adolescent Corona. Although the sign of the estimated coefficient is appropriate, it is not statistically meaningful. This may be because it remains unchanged in all the corresponding years, as the number changed with the five-year interval.

## Conclusion, limitation and future study direction

The aim of this study is to explore the efficacy of selected Asian countries in regulating the spread of adolescent Corona. Country selection is guided by data availability. Specified objectives are explored based on the available information on identified output, input, and exogenous factors for three consecutive years: 2020, 2021, and 2022. Considering the efficacy of a country in regulating adolescent Corona, our study concludes Kazakhstan is the best and Afghanistan is the worst performer. India’s position is not better than Afghanistan's, ranked second on the list. The analysis of SPF divulges that *Vaccinated, Beds, Doctors, Nurses, and Population density* are the statistically meaningful inputs. Notably, panel regression does not help draw a conclusion. Only *vaccinated, doctors and Stringency index* are the influencing factors under panel regression. The Hausman test's conclusions on the suitability of the REM further support the use of the Technical Inefficiency Effects Model (TIEM) in the SPF. The large negative *Log(likelihood)* value and statistical significance of the variance parameters jointly support the application of the Technical Inefficiency Effects Model (TIEM). Contrarily, the exogenous factors that significantly influence (either favourably or adversely) the regulation of adolescents Corona across Asian nations are the *Stringency index (SI), Internet, Urban, Tobacco, and Employment*.

The obtained empirical evidence prompt us to recommend the following policy recommendations:

*Firstly,* vaccination always lowers the risk or, at the very least, controls the severity of the illness. To ensure that all residents have access to the bare minimum in medical care, it is the duty of a nation's government. During a pandemic, every country first immunises the elderly, who were thought to be at high risk, and then immunises adolescents. Therefore, it is the nation's duty to see that every adolescent receives their vaccination on schedule. Only by vaccinating each and every individual can a government minimise the hazards to public health. All people have a fundamental right to receive the Corona vaccination in light of the pandemic scenario. Therefore, if this goal is met, all nations must guarantee that all adolescents receive the complete Corona vaccine.

*Secondly,* an increase in healthcare professionals is always welcome. Therefore, a rise in doctors, nurses, and beds in multispecialty hospitals is a prerequisite for maintaining a nation's health predicament. Therefore, it is always strongly advised that a country adopt positive measures to enhance health staff and health infrastructure, regardless of the present statistics.

*Thirdly,* Asian countries are recognised as densely populated countries. Most Asian countries are either in stage 2 or stage 3 of demographic transition Hussain et al.^73^, which suggests a rapid population increase. Corona is a highly contagious disease that would inevitably spread like wildfire in such conditions. Such infections can only be managed by adopting precautions, including wearing a mask, routinely washing your hands, and being vaccinated. The most important requirements for these nations are adequate medical professionals, appropriate medical infrastructure, and public knowledge of these issues. Thus, it is advised that all of the listed nations adhere to the WHO recommendations for preserving the current health situation.

*Fourthly,* any type of desired consequence for a country is usually acknowledged by an effective government. Therefore, it is advised that all nations, regardless of their existing situation, increase their Stringency index number in order to get better results.

*Fifthly,* the epidemic creates a new normal in which work-from-home opportunities, online drug and grocery shopping, online schooling, online doctor consultations, etc. are all typical occurrences. Therefore, it is the duty of a nation to educate its people about the internet and to offer high-speed network services. In order to accomplish this aim, all nations must take proactive measures.

*Sixthly,* rapid urbanisation is another common phenomenon in Asian countries. It is a particular outcome of a nation's demographic transition period. Rapid industrialization and corresponding rural–urban migration are common traits of Asian countries. Consequently, the governments of such countries need to take appropriate initiatives to provide a minimum standard of living to these migrant people who are living on the outskirts of the metros. Furthermore, rural farm as well as non-farm industrialization is urgently needed to stop rural–urban as well as out-of-country migration. Nations ought to work towards this.

*Seventhly,* employment with a guaranteed salary is the primary input for improving the living standard of citizens. A demographic dividend with a large working-age population is common in Asian countries. The efficient use of this window of opportunity necessitates a sufficient supply of raw materials and energy. Only by fostering both work and self-employment options will this demographic dividend be controlled, resulting in rapid growth and development.

*Finally,* in Asian nations, adolescents’ tobacco intoxication is highly prevalent. The only restrictions that can prohibit its expansion are laws and regulations. Regular anti-intoxication counselling in schools and colleges may have positive consequences. Therefore, authorities should implement such measures to prevent intoxication among adolescents.

Coming to the shortcomings of this study, we can say that it was conducted by considering selected Asian countries based on data availability. Asian countries experience a dichotomous economy where the rural economy is significantly backward from the urban sector. Thus, aggregate statistics are not well accepted for such analysis. A more appropriate conclusion may be obtained by performing the analysis for rural and urban economies separately. However, the non-availability of such statistics restricts us to perform this. Additionally, the proportion of female political leaders is a good indicator of female political engagement. Again, the absence of such data forces us to estimate FPP based on the proportion of women in the lower house of parliament, which is prone to change during a five-year period. The outcome thus loses statistical significance. Technically, Battese and Coelli^39^, model is considered to be time-invariant. However, as we considered a very short panel for our analysis, this limitation had little impact on the final score. Moreover, the "adolescent suicidal mortality rate (per 100,000) and tobacco usage in adolescents (%)" data are used to illustrate the behavioural changes among adolescents in Asian countries. In this instance, "number of adolescents suffering from mental depression" would be a more pertinent metric. However, the lack of data prevents us from taking this variable into account. Additionally, the listed two variables are taken into account for 2019 to 2023 while studying the modifiable change in adolescents' behaviour. The results could change if more time periods were included. Again, the lack of data forced us to focus exclusively on those five time points.

Regarding the study's future direction, we want to emphasise that, subject to the availability of pertinent data, we are serious about expanding the study to include all Asian nations as well as the rural and urban economies separately. We also prefer to take into account a few other factors, such as female political leaders, standard of living, number adolescents’ mental depression cases, etc., that might affect how well a nation controls an infectious disease. Additionally, we intend to continue the study for a longer period of time to capture pre-and-post pandemic scenario.

### Supplementary Information

Supplementary Information.

## Data Availability

All of the secondary data sources used in the study are cited in the text. Please refer to Table 1 for further details.

## References

[1] Ramraj, V. V. (Ed.). *Covid-19 in Asia: Law and policy contexts*. Oxford University Press, (2020).

[2] Chakraborty I, Maity P (2020). COVID-19 outbreak: Migration, effects on society, global environment and prevention. Sci. Total Environ..

[3] Bhagat RB, Reshmi RS, Sahoo H, Roy AK, Govil D (2020). The COVID-19, migration and livelihood in India: Challenges and policy issues. Migration Letters.

[4] Jomo KS, Chowdhury A (2020). Covid-19 pandemic recession and recovery. Development.

[5] Moore RC, Lee AY, Hancock JT, Halley MC, Linos E (2021). Age-related differences in experiences with social distancing at the onset of the COVID-19 pandemic: A computational and content analytic investigation of natural language from a social media survey. JMIR human factors.

[6] WHO,. COVID-19 disease in children and adolescents: Scientific brief. Retrieved from https://www.who.int/publications/i/item/WHO-2019-nCoV-Sci_Brief-Children_and_adolescents-2021.1. Accessed on 23 April 2023 (2021).

[7] Zhou Y, Yang Q, Chi J, Dong B, Lv W, Shen L, Wang Y (2020). Comorbidities and the risk of severe or fatal outcomes associated with coronavirus disease 2019: A systematic review and meta-analysis. Int. J. Infect. Dis..

[8] Turke PW (2021). Five reasons COVID-19 is less severe in younger age-groups. Evol. Med. Public Health.

[9] Sinaei R, Pezeshki S, Parvaresh S, Sinaei R (2021). Why COVID-19 is less frequent and severe in children: A narrative review. World J. Pediatr..

[10] NWHN. How does COVID-19 affect different age groups? Retrieved from https://nwhn.org/how-does-covid-19-affect-different-age-groups/. Accessed on 23 April 2023 (2020).

[11] Jester N, Kang P (2021). COVID-19 pandemic: Is teenagers’ health in crisis? An investigation into the effects of COVID-19 on self-reported mental and physical health of teenagers in secondary education. Public Health Pract..

[12] Allabadi H, Dabis J, Aghabekian V, Khader A, Khammash U (2020). Impact of COVID-19 lockdown on dietary and lifestyle behaviours among adolescents in Palestine. Dyn Human Health.

[13] Xiang M, Zhang Z, Kuwahara K (2020). Impact of COVID-19 pandemic on children and adolescents' lifestyle behavior larger than expected. Prog. Cardiovasc. Dis..

[14] De Figueiredo, C. S., Sandre, P. C., Portugal, L. C. L., Mázala-de-Oliveira, T., da Silva Chagas, L., Raony, Í., ... & Bomfim, P. O. S. COVID-19 pandemic impact on children and adolescents' mental health: Biological, environmental, and social factors. *Prog. Neuro-Psychopharmacol. Biol. Psychiatry*, *106*, 110171 (1996). 10.1016/j.pnpbp.2020.110171 PMC7657035 33186638

[15] Mia, M. A., & Griffiths, M. D. Can South Asian countries cope with the mental health crisis associated with COVID-19?. *Int. J. Mental Health Addict.* 1–10 (2021). 10.1007/s11469-021-00491-5 PMC7886190 33613132

[16] Gazmararian J, Weingart R, Campbell K (2021). Impact of COVID-19 pandemic on the mental health of students from 2 semi-rural high schools in Georgia. J Sch Health..

[17] Magson NR, Freeman JYA, Rapee RM (2021). Risk and protective factors for prospective changes in adolescent mental health during the COVID-19 pandemic. J Youth Adolesc.

[18] Temple JR, Baumler E, Wood L, Guillot-Wright S, Torres E, Thiel M (2022). The impact of the COVID-19 pandemic on adolescent mental health and substance use. J. Adolescent Health.

[19] Kompaniyets, L., Bull-Otterson, L., Boehmer, T. K., Baca, S., Alvarez, P., Hong, K., ... & Saydah, S. Post–COVID-19 symptoms and conditions among children and adolescents—United States, March 1, 2020–January 31, 2022. *Morbidity Mortality Weekly Report*, *71*(31), 993 (2022). 10.15585/mmwr.mm7131a3 PMC9368731 35925799

[20] Melo, M. M., Neta, M. M. R., Neto, A. R. S., Carvalho, A. R. B., Magalhães, R. L. B., Valle, A. R. M. C., & Freitas, D. R. J. Symptoms of COVID-19 in children. *Braz. J. Med. Biol. Res.*, *55*, e12038 (2022). 10.1590/1414-431X2022e12038 PMC9200047 35703681

[21] Crook, H., Raza, S., Nowell, J., Young, M., & Edison, P. Long covid—mechanisms, risk factors, and management. *bmj*, *374* (2021). 10.1136/bmj.n1648 34312178

[22] Douglas, M., Katikireddi, S. V., Taulbut, M., McKee, M., & McCartney, G. Mitigating the wider health effects of covid-19 pandemic response. *Bmj*, *369*, (2020). 10.1136/bmj.m1557 PMC7184317 32341002

[23] Lopez-Leon S, Wegman-Ostrosky T, Perelman C, Sepulveda R, Rebolledo PA, Cuapio A, Villapol S (2021). More than 50 long-term effects of COVID-19: A systematic review and meta-analysis. Scientific reports.

[24] McMichael, T. M., Currie, D. W., Clark, S., Pogosjans, S., Kay, M., Schwartz, N. G., & Duchin, J. S. Epidemiology of COVID-19 in a long-term care facility in King County, Washington. *N. Engl. J. Med.* *382*(21), 2005–2011 (2020). 10.1056/NEJMoa2005412 PMC7121761 32220208

[25] Cohen-Mansfield J, Meschiany G (2022). Direct and indirect effects of COVID-19 on long-term care residents and their family members. Gerontology.

[26] Mantovani A, Rinaldi E, Zusi C, Beatrice G, Saccomani MD, Dalbeni A (2021). Coronavirus disease 2019 (COVID-19) in children and/or adolescents: A meta-analysis. Pediatric research.

[27] Wang, J., Aaron, A., Baidya, A., Chan, C., Wetzler, E., Savage, K., & Kang, Y. Gender differences in psychosocial status of adolescents during COVID-19: a six-country cross-sectional survey in Asia Pacific. *BMC Public Health*, *21*(1), 1–18 (2021). 10.1186/s12889-021-12098-5 PMC8568363 34736426

[28] Chua, G. T., Xiong, X., Choi, E. H., Han, M. S., Chang, S. H., Jin, B. L., & Kwan, M. Y. W. COVID-19 in children across three Asian cosmopolitan regions. *Emerg. Microbes Infect.* *9*(1), 2588–2596 (2020). 10.1080/22221751.2020.1846462 PMC7723019 33138739

[29] Nunamaker TR (1983). Measuring routine nursing service efficiency: a comparison of cost per patient day and data envelopment analysis models. Health Serv Res.

[30] Sherman HD (1984). Hospital efficiency measurement and evaluation: empirical test of a new technique. Med. Care.

[31] Fernandez, R. M. Gross domestic product and health. *Good Health and Well-Being*, 237–245 (2020).

[32] Murray C, Frenk J (2001). World Health Report 2000: A step towards evidence-based health policy. Lancet.

[33] Sankar, D., & Kathuria, V. Health system performance in rural India: efficiency estimates across states. *Econ. Polit. Weekly*, 1427–1433 (2004).

[34] Kathuria V, Sankar D (2005). Inter-state disparities in health outcomes in rural India: An analysis using a stochastic production frontier approach. Dev. Policy Rev..

[35] Farrell MJ (1957). The measurement of productive efficiency. J. R. Stat. Soc. Ser. A: Stat. Soc..

[36] Evans DB, Tandon A, Murray CJ, Lauer JA (2000). The comparative efficiency of national health systems in producing health: An analysis of 191 countries. World Health Organ...

[37] Murray, C. J. L, Frenk, J., World Health Organization. A WHO framework for health system performance assessment. *Global Programme on Evidence for Health Policy*, (1999).

[38] Maity S, Barlaskar UR (2022). Women's political leadership and efficiency in reducing COVID-19 death rate: An application of technical inefficiency effects model across Indian states. Socio-Econ. Plan. Sci..

[39] Battese GE, Coelli TJ (1995). A model for technical inefficiency effects in a stochastic frontier production function for panel data. Empir. Econ..

[40] World Health Organization. *The second decade: Improving adolescent health and development* (No. WHO/FRH/ADH/98.18 Rev. 1). World Health Organization (2001).

[41] Greenberg M, Schneider D (2023). Population density: What does it really mean in geographical health studies?. Health & Place.

[42] Braveman, P., & Gottlieb, L. The social determinants of health: it's time to consider the causes of the causes. *Public Health Rep.* *129*(1_suppl2), 19–31 (2014). 10.1177/00333549141291S206 PMC3863696 24385661

[43] Mulatu, M. S., & Schooler, C. Health: reciprocal effects and mediating mechanisms. *J Health Soc. Behav*. *43*(1), (2002). 11949195

[44] Kumbhakar SC (1991). Estimation of technical inefficiency in panel data models with firm-and time-specific effects. Econ. Lett..

[45] Stevenson RE (1980). Likelihood functions for generalized stochastic frontier estimation. J. Econom..

[46] Coelli, T. J. *A guide to FRONTIER version 4.1: A computer program for stochastic frontier production and cost function estimation* (Vol. 7, pp. 1–33). CEPA Working papers, (1996).

[47] Baltagi, B. H. Econometric Analysis of Panel Data, Wiley. *West Sussex, England*, (2005).

[48] Pesaran MH (2006). Estimation and inference in large heterogeneous panels with a multifactor error structure. Econometrica.

[49] Chisholm D, Evans DB (2010). Improving health system efficiency as a means of moving towards universal coverage. World health report.

[50] Maity S, Neogi C (2014). Production of tea in Assam and West Bengal: Technical inefficiency effects. Artha Vijnana.

[51] Gandy S (2011). Perspective: prevention is better than cure. Nature.

[52] Jan Zakri JM, Rabun MN, Mohamad Nazir MSR (2021). Prevention is better than cure: A case of parents’ decisions of children vaccinations. Voice of Academia (VOA).

[53] Giubilini, A., Savulescu, J., & Wilkinson, D. COVID-19 vaccine: vaccinate the young to protect the old?. *J. Law Biosci.**7*(1), lsaa050 (2020). 10.1093/jlb/lsaa050 PMC7337759 32959006

[54] Mascellino, M. T., Di Timoteo, F., De Angelis, M., & Oliva, A. Overview of the main anti-SARS-CoV-2 vaccines: mechanism of action, efficacy and safety. *Infect. Drug Resistance*, 3459–3476 (2021). 10.2147/IDR.S315727 PMC8418359 34511939

[55] Di Lorenzo G, Di Trolio R (2020). Coronavirus disease (COVID-19) in Italy: analysis of risk factors and proposed remedial measures. Front. Med..

[56] Xin, X., Li, S. F., Cheng, L., Liu, C. Y., Xin, Y. J., Huang, H. L., & Feng, L. Government intervention measures effectively control COVID-19 epidemic in Wuhan, China. *Curr. Med. Sci.**41*, 77–83 (2021). 10.1007/s11596-021-2321-6 PMC7881908 33582909

[57] Biswas N, Mustapha T, Khubchandani J, Price JH (2021). The nature and extent of COVID-19 vaccination hesitancy in healthcare workers. J. Community Health.

[58] GÜNER, H. R., Hasanoğlu, I., & Aktaş, F. Evaluating the efficiency of public policy measures against COVID-19. *Turkish J. Med. Sci.**51*(7), 3229–3237 (2021). 10.3906/sag-2106-301 PMC8771047 34391324

[59] Oran DP, Topol EJ (2020). Prevalence of asymptomatic SARS-CoV-2 infection: A narrative review. Ann. Internal Med.

[60] Neville FG, Templeton A, Smith JR, Louis WR (2021). Social norms, social identities and the COVID-19 pandemic: Theory and recommendations. Soc. Personality Psychol. Compass.

[61] Alam MZ (2021). Is population density a risk factor for communicable diseases like COVID-19? A case of Bangladesh. Asia Pacific J. Public Health.

[62] Sharma GD, Talan G, Srivastava M, Yadav A, Chopra R (2020). A qualitative enquiry into strategic and operational responses to Covid-19 challenges in South Asia. J. Public Affairs.

[63] Burnell P (1994). Good government and democratization: A sideways look at aid and political conditionality. Democratization.

[64] Dias A, Scavarda A, Silveira H, Scavarda LF, Kondamareddy KK (2021). The online education system: COVID-19 demands, trends, implications, challenges, lessons, insights, opportunities, outlooks, and directions in the work from home. Sustainability.

[65] Rachmawati R, Choirunnisa U, Pambagyo ZA, Syarafina YA, Ghiffari RA (2021). Work from home and the use of ICT during the COVID-19 pandemic in Indonesia and its impact on cities in the future. Sustainability.

[66] Libert M, Le Cam F, Domingo D (2022). Belgian journalists in lockdown: Survey on employment and working conditions and representations of their role. J. Stud..

[67] Pinchoff J, Friesen EL, Kangwana B, Mbushi F, Muluve E, Ngo TD, Austrian K (2021). How has COVID-19-related income loss and household stress affected adolescent mental health in Kenya?. J. Adolescent Health.

[68] Salama AH, Ragab DA, Abdel-Moneim NM (2023). Urban spaces as a positive catalyst during pandemics: Assessing the community’s well-being by using artificial intelligence techniques. Ain Shams Eng. J..

[69] Baker, J., Krishnan, N., Abroms, L. C., & Berg, C. J. The impact of tobacco use on COVID-19 outcomes: a systematic review. *J. Smoking Cessation*, (2022). 10.1155/2022/5474397 PMC8777389 35126740

[70] Kalkhoran S, Benowitz NL, Rigotti NA (2018). Prevention and treatment of tobacco use: JACC health promotion series. J. Am. Coll. Cardiol..

[71] Warren, C. W., Riley, L., Asma, S., Eriksen, M. P., Green, L., Blanton, C., & Yach, D. Tobacco use by youth: a surveillance report from the Global Youth Tobacco Survey project. *Bull. World Health Organ.* *78*(7), 868–876 (2000). PMC2560802 10994259

[72] Calihan JB, Levy S (2022). Coronavirus disease pandemic and adolescent substance use. Curr. Opin. Pediatr..

[73] Hussain, A., Cassen, R., & Dyson, T. Demographic transition in Asia and its consequences. *Sociol. Ageing*, 321–333 (2009).

