# Supplementary material for: Technical efficiency and its determinants in regulating adolescents’ coronavirus infection across Asian countries
Source: Sci Rep. 2023 Nov 1;13:18841. doi: 10.1038/s41598-023-45442-3 (PMC10620206; doi:10.1038/s41598-023-45442-3)
Supplement: Supplementary file 1 — Supplementary Information. [file 41598_2023_45442_MOESM1_ESM.docx]

**Appendices**

**Table-A.1: Summary statistics for output and input variables of the frontier model**

| **Variables** | **Mean** | **SD** | **Min** | **Max** |
| --- | --- | --- | --- | --- |
| Number of adolescent COVID-19 positive cases | 408135.60 | 1033242.00 | 49.1 | 6254974 |
| $\frac{1}{Y}$ | 0.00 | 0.00 | 1.60E-07 | 0.020367 |
| $\ln\left(\frac{1}{Y}\right)$ | -11.21 | 2.29 | -15.65 | -3.89 |
| Fully vaccinated (%) | 53.91 | 23.03 | 8 | 90.9 |
| Hospital beds per thousand | 2.70 | 2.84 | 0.3 | 13.15 |
| Per-capita GDP | 7897.13 | 10331.43 | 426.03 | 40805.19 |
| Doctor per 1000 | 1.62 | 1.04 | 0.21 | 4.07 |
| Nurses and Midwives (per 1000) | 3.04 | 2.18 | 0.3962 | 11.948 |
| Population density (people per sq. km of land area) | 267.64 | 275.60 | 6.86 | 1286.17 |
| Female Political Participation (% in lower parliament) | 17.80 | 8.19 | 4.7 | 32.7 |
| Stringency index | 51.47 | 18.75 | 11.42 | 77.56 |
| Internet use (%) | 63.27 | 22.55 | 17.07 | 96.75 |
| Urbanisation (%) | 54.36 | 24.78 | 18.59 | 92.67 |
| Tobacco use in adolescents (%) | 13.62 | 9.06 | 1.45 | 36.85 |
| Regular wage emp. (%) | 56.56 | 20.66 | 15.67 | 90.17 |

*Source: Authors’ own calculation based on secondary data*
